# Performance evaluation of the Cobas c 703 analytical unit as part of Cobas Pro integrated solutions using LED as the sole photometric light source

**DOI:** 10.1016/j.plabm.2025.e00482

**Published:** 2025-06-03

**Authors:** Peter Findeisen, Inger Brandt, Frederic Winnock, Jan Furrer, Kai Klopprogge

**Affiliations:** a Medizinisches Versorgungszentrum (MVZ) Labor Dr. Limbach & Kollegen eGbR, Heidelberg, Germany; b Onze Lieve Vrouwziekenhuis (OLV), Aalst, Belgium; c Algemeen Stedelijk Ziekenhuis (ASZ), Aalst, Belgium; d Roche Diagnostics International Ltd, Rotkreuz, Switzerland; e Roche Diagnostics GmbH, Mannheim, Germany

**Keywords:** Clinical chemistry, LED, Automation, Evaluation, Photometric light source

## Abstract

**Background:**

Most commercially available clinical chemistry systems today rely on a halogen light source to power photometric measurements, lasting only up to ∼750 h before replacement is required. Halogen lamps will soon be phased out in favor of new longer-lasting light-emitting diodes (LED) in commercial devices.

**Methods:**

We compared the analytical performance of the new Cobas® c 703 analytical unit (LED; Cobas Pro integrated solutions) versus the Cobas c 701/c 702 modules (halogen light source; Cobas 8000 modular analyzer series; all Roche Diagnostics International Ltd). At two evaluation sites, precision of the c 703 analytical unit was evaluated and method comparison experiments comparing the c 703 analytical unit versus c 701/c 702 modules were performed.

**Results:**

Results from 34 selected applications showed robust precision of the c 703 analytical unit (repeatability, intermediate precision, and reproducibility: 80/138 coefficients of variation <1 %, 60/138 < 1 %, and 48/69 < 2 %, respectively). Method comparison experiments with 31 applications showed good comparability between c 703 and c 701/c 702 using LED versus halogen light sources, respectively (median slope = 1.00; median bias = 0.2 %; median Pearson's correlation coefficient = 0.997).

**Conclusions:**

In addition to the newly introduced LED light source having a longer lifetime (10,000 h; data not shown), the c 703 analytical unit demonstrated equivalent analytical performance to that of the c 701/c 702 modules using halogen lamps as the light source as part of high-throughput clinical chemistry analyzers.

## Highlights

• This study compared Cobas® c 703 using LED vs Cobas c 701/c 702 using halogen lamps.
• LED have been shown to last longer than halogen as a light source.
• The c 703 analytical unit using LED demonstrated robust precision.
• Good comparability was observed between analytical units/light sources.
• c 703 shows equivalent analytical performance vs c 701/c 702.

## 1 Introduction

Halogen lamps have been the light source of choice for automated clinical chemistry analyzers for decades, providing a reliable light source from 340 to 800 nm for a wide variety of photometric assays. Light-emitting diodes (LED) have replaced these conventional light sources in homes, televisions, and car headlights, and provide brighter, longer-lasting light (10,000 h; data on file) with reduced power intake and heat production compared with halogen lamps. In an automated clinical chemistry setting, a LED light source was first introduced to support the halogen lamp at 340 nm with the Siemens Atellica CH 930 Analyzer. The newly developed high-throughput Cobas® c 703 analytical unit, part of Cobas Pro integrated solutions (both Roche Diagnostics International Ltd, Rotkreuz, Switzerland), relies on LED as the sole light source from 340 to 800 nm. Here, we report analytical performance in terms of precision and comparability of the c 703 analytical unit using LED as the sole light source versus the Cobas c 701 and c 702 modules (Cobas 8000 modular analyzer series; all Roche Diagnostics International Ltd, Rotkreuz, Switzerland) using halogen lamps as the photometer light source.

## 2 Materials and methods

This study was conducted at two sites in Europe (Aalst, Belgium, and Heidelberg, Germany). Analytical performance and functionality of the c 703 analytical unit were assessed using a 21-day precision protocol and routine-like method comparison experiments. Two Cobas Pro integrated solution configurations were tested using the c 703 analytical unit with LED as the sole light source: one configuration included one Cobas ISE neo analytical unit, two c 703 analytical units, and one Cobas e 801 analytical unit; the other configuration included Cobas ISE neo, c 703, Cobas c 503, and e 801 analytical units (one each) (all products Roche Diagnostics International Ltd, Rotkreuz, Switzerland). From various ion-selective electrode (ISE) and immunochemistry applications, 34 applications were selected and tested on the c 703 analytical unit for precision and 31 applications for comparability of the c 703 (Cobas Pro integrated solutions) versus the c 701/c 702 modules (8000 modular analyzer series; Supplementary Table 1). The applications were selected prior to the start of the study to cover all aspects of the existing clinical chemistry menu (comprising all available tests on the c 703 analytical unit, based on photometric detection), like sample and reagent pipetting volume, reagent viscosity, and detection wavelength. The selection also encompassed ∼80 % of the routinely used clinical chemistry applications in the core laboratory of the participating evaluation sites for routine-like method comparisons.

### 2.1 Precision

Precision experiments based on the Clinical and Laboratory Standards Institute EP05-A3 protocol were conducted, with two runs per day over a 21-day period for 34 selected clinical chemistry applications representing a typical routine panel of assays and covering differences in assay protocols. Pooled control materials were used to minimize the potential influence of sample handling between sites and operators; two control samples were measured per application (quality control [QC] low and QC high concentration).

Coefficients of variation (CVs) for repeatability (within-run precision), intermediate precision (within-laboratory precision), and reproducibility (across-laboratory precision) were calculated and compared with pre-defined acceptance criteria.

### 2.2 Routine simulation method comparison

At the two sites, routine simulation method comparison experiments [1] using residual samples were conducted to evaluate overall functionality under routine-like conditions and comparability of the c 703 analytical unit combined with Cobas ISE neo, c 503, and e 801 analytical units (Cobas Pro) versus c 701 and c 702 modules combined with the e 801 analytical unit (8000 modular analyzers). Among tests covering ISE, clinical chemistry, and immunochemistry, in total 25 analytes with 31 applications were assessed on the c 703 analytical unit. To introduce variability within and across sites, testing was performed for 12 and 14 days, respectively, per site. The analyte concentration ranges covered per assay reflected those of the individual sites’ routine samples at that time. Pre-collected or spiked samples were not included.

Routine workloads (up to 1300 samples) were replicated and reprocessed, in part or total, on Cobas Pro integrated solutions using WebCAEv (a Code of Federal Regulations Title 21 Part 11 compliant electronic data capture software developed and validated for Roche-sponsored studies [2]) to capture routine analyzer requests via an electronic file from the Laboratory Information Systems (Host). This experimental design enabled laboratory personnel to assess overall system functionality, including sample and reagent handling, data flagging and alarm generation, and flow of workload processing [1]. Routine residual samples were stored up to several days before remeasurement on Cobas Pro integrated solutions.

Results per sample were compared with those previously generated during routine operation; data pairs were automatically evaluated using Passing-Bablok regression [3], and slopes, intercepts, and correlations for method comparisons were calculated and compared with pre-defined acceptance criteria.

## 3 Results

### 3.1 Precision

**Fig. 1:**
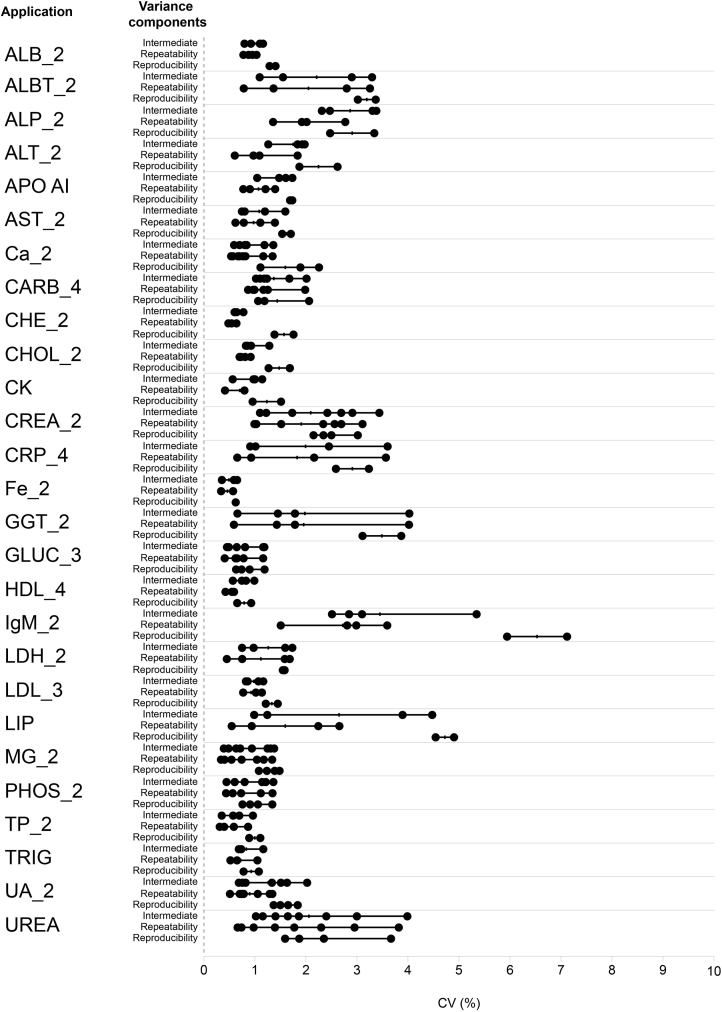
Variability of precision CVs for the c 703 analytical unit. ALB, albumin; ALBT, Tina-quant albumin; ALP, alkaline phosphatase; ALT, alanine aminotransferase; APO AI, apolipoprotein A-1; AST, aspartate aminotransferase; Ca, calcium; CARB, carbamazepine; CHE, cholinesterase; CHOL, cholesterol; CK, creatine kinase; CREA, creatinine; CRP, C-reactive protein; CV, coefficient of variation; Fe, iron; GGT, γ-glutamyltransferase; GLUC, glucose; HDL, high-density lipoprotein; IgM, immunoglobulin; LDH, lactate dehydrogenase; LDL, low-density lipoprotein; LIP, lipase; MG, magnesium; PHOS, phosphate; TP, total protein; TRIG, triglycerides; UA, uric acid.

In total, 345 CVs were calculated, including repeatability (n = 138), intermediate precision (n = 138), and reproducibility (n = 73) (Supplementary Table 2). For repeatability, intermediate precision, and reproducibility, 80/138 CVs were <1 %, 60/138 CVs were <1 %, and 48/69 CVs were <2 %, respectively (Supplementary Table 2, Fig. 1).

### 3.2 Routine simulation method comparison

More than 135,000 data pairs were included in the regression analysis for the 25 analytes with 31 applications assessed on the c 703 analytical unit versus c 701/c 702 modules across both evaluation sites.

The 31 method comparisons regression analysis generated a median slope of 1.00, ranging from 0.85 (Phosphate [urine]) to 1.12 (C-reactive protein). The median bias at the medical decision point was 0.2 %, ranging from −6 % (Urea [urine]) to 11 % (Aspartate aminotransferase). The median Pearson's correlation coefficient was 0.997, ranging from 0.945 (Calcium) to 1.000 (Glucose [urine]) (Table 1). Supplementary Fig. 1 shows Passing-Bablok regression and Bland-Altman plots of the two applications with the highest bias in slope (CRP) and intercept (CHE). CRP shows a disproportional amount of data in the lower concentration range, resulting in a regression slope that does not match most of the higher concentration data pairs. Bias between evaluation sites is also visible. The relatively high y-intercept (absolute value 71 U/L) for CHE is due to the high concentration range of the assay; the normalized difference is between 6 and −8 % over the whole concentration range.

**Table 1:** Summary of routine method comparisons using the c 703 analytical unit versus the c 701 and c 702 modules.

Application	Study Unit	Specimen	N	Min X	Max X	Slope	Intercept	Pearson's r
ALP_2	U/L	Serum/Plasma	7426	21.0	1159	0.99	0.87	0.9985
ALT_2	U/L	Serum/Plasma	10023	5.00	611	1.00	0.90	0.9945
AST_2	U/L	Serum/Plasma	8687	5.30	549	1.02	1.77	0.9845
CHE_2	U/L	Serum/Plasma	427	2261	13458	0.99	−70.8	0.9977
CK	U/L	Serum/Plasma	2127	11	1797	0.99	0	0.9996
GGT_2	U/L	Serum/Plasma	10579	4.00	1129	1.04	−0.47	0.9977
LDH_2	U/L	Serum/Plasma	4421	55.0	929	0.98	−0.67	0.9972
LIP	U/L	Serum/Plasma	2588	5.90	292	1.00	−0.83	0.9930
ALB_2	g/L	Serum/Plasma	1592	9.70	54.3	0.92	5.32	0.9602
CHOL_2	mmol/L	Serum/Plasma	6777	1.14	16.8	1.02	−0.05	0.9967
CREA_2	μmol/L	Serum/Plasma	14045	15.7	1581	1.02	1.04	0.9986
GLUC_3	mmol/L	Serum/Plasma	6428	1.18	40.9	1.01	−0.01	0.9984
HDL_4	mmol/L	Serum/Plasma	5450	0.319	3.68	0.95	0.029	0.9967
LDL_3	mmol/L	Serum/Plasma	4119	0.10	8.00	1.00	−0.01	0.9979
TP_2	g/L	Serum/Plasma	3951	37.70	110	1.01	−1.33	0.9635
TRIG	mmol/L	Serum/Plasma	6258	0.31	9.72	1.00	0.05	0.9980
UA_2	μmol/L	Serum/Plasma	7722	53.6	1476	1.01	1.53	0.9978
UREA	mmol/L	Serum/Plasma	7852	1.08	39.5	0.98	0.18	0.9975
Ca_2	mmol/L	Serum/Plasma	7976	1.31	3.07	1.00	0.01	0.9456
MG_2	mmol/L	Serum/Plasma	1449	0.190	1.30	0.99	−0.00	0.9716
PHOS_2	mmol/L	Serum/Plasma	4570	0.24	4.78	0.99	0.03	0.9951
Fe_2	μmol/L	Serum/Plasma	4565	1.32	79.8	1.00	−0.08	0.9988
IgM_2	g/L	Serum/Plasma	376	0.260	6.18	0.95	0.01	0.9957
CRP_4	mg/L	Serum/Plasma	7017	0.61	340	1.12	−0.15	0.9976
CREA_2	μmol/L	Urine	756	604	47648	1.05	48.0	0.9969
GLUC_3	mmol/L	Urine	81	0.12	33.7	1.00	0.00	0.9998
UA_2	μmol/L	Urine	23	476	4095	0.95	−10.1	0.9981
UREA	mmol/L	Urine	32	34.0	313	0.93	2.21	0.9864
MG_2	mmol/L	Urine	16	0.72	3.15	1.04	−0.15	0.9849
PHOS_2	mmol/L	Urine	16	3.12	19.17	0.85	1.31	0.9819
ALBT_2	mg/L	Urine	284	8.0	380	1.03	−0.76	0.9985

ALB, albumin; ALBT, Tina-quant albumin; ALP, alkaline phosphatase; ALT, alanine aminotransferase; APO AI, apolipoprotein A-1; AST, aspartate aminotransferase; Ca, calcium; CARB, carbamazepine; CHE, cholinesterase; CHOL, cholesterol; CK, creatine kinase; CREA, creatinine; CRP, C-reactive protein; Fe, iron; GGT, γ-glutamyltransferase; GLUC, glucose; HDL, high-density lipoprotein; IgM, immunoglobulin; LDH, lactate dehydrogenase; LDL, low-density lipoprotein; LIP, lipase; MG, magnesium; PHOS, phosphate; TP, total protein; TRIG, triglycerides; UA, uric acid.

## 4 Discussion

In this study, the novel c 703 analytical unit (part of Cobas Pro integrated solutions) for clinical chemistry testing was assessed extensively for overall analytical performance and functionality versus data generated on the c 701/c 702 modules (8000 modular analyzer series) via precision and routine-like method comparison experiments. Our findings demonstrated that under routine-like conditions, the c 703 analytical unit using a sole LED light source had robust precision and demonstrated comparable results with the c 701 and c 702 modules using halogen lamps as the light source, supporting its implementation in routine clinical laboratory practice.

The obvious benefits of using a LED light source for photometric testing, like durability, efficiency, and practicability, were shown to not impair result quality. The lifetime of the c 703 analytical unit is 10,000 h (data on file), compared with ∼750 h for traditional halogen light sources. High-quality analytical performance is essential, and the precision and method comparison data demonstrated the reliability of the c 703 analytical unit. The demonstrated precision and comparability versus the c 701 and c 702 modules do not differ from existing clinical chemistry analyzers.

When comparing results from the c 703 analytical unit with results from routine, randomly selected samples at two study sites, the 34 applications chosen for precision testing and the 31 applications used during routine method comparison showed consistent results.

In conclusion, the c 703 analytical unit as part of Cobas Pro integrated solutions demonstrated robust analytical performance using LED as the sole photometric light source, supporting its implementation in routine clinical laboratory practice and the potential to further consolidate in vitro diagnostic testing in high-throughput laboratories.

## CRediT authorship contribution statement

**Peter Findeisen:** Writing – review & editing, Writing – original draft, Formal analysis, Data curation. **Inger Brandt:** Writing – review & editing, Writing – original draft, Formal analysis, Data curation. **Frederic Winnock:** Writing – review & editing, Writing – original draft, Formal analysis, Data curation. **Jan Furrer:** Writing – review & editing, Writing – original draft, Formal analysis, Data curation, Conceptualization. **Kai Klopprogge:** Writing – review & editing, Writing – original draft, Formal analysis, Data curation, Conceptualization.

## Ethical approval

The study was approved by the institutional review boards or equivalent committees for the respective study sites: Landesärztekammer Baden-Württemberg (Heidelberg: approval no. F-2023-051) and the A.S.Z. Commisie voor Medische Ethiek (Aalst, identifier Cobas Pro ISE). The study complied with all relevant national regulations and institutional policies and was performed in accordance with the principles of the Declaration of Helsinki.

## Funding

This study was funded by 10.13039/100016545Roche Diagnostics GmbH (Mannheim, Germany).

## Declaration of competing interest

**Peter Findeisen, Inger Brandt and Frederic Winnock** report provision of funding for the present study from 10.13039/100016545Roche Diagnostics to their institute. **Jan Furrer** is an employee of 10.13039/100016545Roche Diagnostics International Ltd (Rotkreuz, Switzerland) and holds stocks in F. Hoffmann-La Roche Ltd. **Kai Klopprogge** is an employee of Roche Diagnostics GmbH (Mannheim, Germany) and holds stocks in F. Hoffmann-La Roche Ltd.

## Data Availability

The data that has been used is confidential.
