# Supplementary material for: Performance evaluation of the Cobas c 703 analytical unit as part of Cobas Pro integrated solutions using LED as the sole photometric light source
Source: Pract Lab Med. 2025 Jun 3;46:e00482. doi: 10.1016/j.plabm.2025.e00482 (PMC12174593; doi:10.1016/j.plabm.2025.e00482)
Supplement: Multimedia component 1 [file mmc1.docx]

**Supplementary Material**

**Performance evaluation of the Cobas c 703 analytical unit as part of Cobas Pro integrated solutions using LED as the sole photometric light source**

Peter Findeisen, Inger Brandt, Frederic Winnock, Jan Furrer, Kai Klopprogge

**Supplementary Table 1** Applications and corresponding wavelengths used.

| Experiment* | Application | Application Code | Wavelength Main (nm) | Wavelength Sub (nm) |
| --- | --- | --- | --- | --- |
| P, MC | ALB_2 | 20090 | 570 | 505 |
| P, MC | ALBT_2 | 20061 | 340 | 700 |
| P, MC | ALP_2 | 20110 | 450 | 480 |
| P, MC | ALTP_2 | 20140 | 340 | 700 |
| P | APO AI | 20190 | 340 | 700 |
| P, MC | ASTP_2 | 20230 | 340 | 700 |
| P, MC | Ca_2 | 20340 | 340 | 376 |
| P | Ca_2 | 20341 | 340 | 376 |
| P | CARB_4 | 20351 | 546 | 800 |
| P, MC | CHE_2 | 20370 | 415 | 700 |
| P, MC | CHOL_2 | 20411 | 505 | 700 |
| P, MC | CK | 20420 | 340 | 546 |
| P, MC | CREJ_2 | 20470 | 505 | 570 |
| P, MC | CREJ_2 | 20471 | 505 | 570 |
| P, MC | CRP_4 | 20500 | 570 | 800 |
| P, MC | Fe_2 | 20770 | 570 | 700 |
| P, MC | GGT_2 | 20600 | 415 | 700 |
| P, MC | GLUC_3 | 20630 | 340 | 700 |
| P, MC | GLUC_3 | 20631 | 340 | 700 |
| P, MC | HDLC_4 | 20710 | 600 | 700 |
| P, MC | IgM_2 | 20750 | 340 | 700 |
| P, MC | LDHI_2 | 20810 | 340 | 700 |
| P, MC | LDLC_3 | 20820 | 600 | 700 |
| P, MC | LIPC | 20850 | 570 | 700 |
| P, MC | MG_2 | 20890 | 600 | 505 |
| P, MC | MG_2 | 20891 | 600 | 505 |
| P, MC | PHOS_2 | 20990 | 340 | 700 |
| P, MC | PHOS_2 | 20991 | 340 | 700 |
| P, MC | TP_2 | 21110 | 546 | 700 |
| P, MC | TRIG | 21130 | 505 | 700 |
| P, MC | UA_2 | 21170 | 546 | 700 |
| P, MC | UA_2 | 21171 | 546 | 700 |
| P, MC | UREAL | 21190 | 340 | 700 |
| P, MC | UREAL | 21191 | 340 | 700 |

*P indicates precision experiment; MC indicates routine method comparison experiment.
ALB, albumin; ALBT, Tina-quant albumin; ALP, alkaline phosphatase; ALT, alanine aminotransferase; ALTP, alanine aminotransferase according to IFCC; APO AI, apolipoprotein A‑1; ASTP, aspartate aminotransferase according to IFCC; Ca, calcium; CARB, carbamazepine; CHE, cholinesterase; CHOL, cholesterol; CK, creatine kinase; CREJ, creatinine Jaffé; CRP, C-reactive protein; Fe, iron; GGT, γ-glutamyltransferase; GLUC, glucose; HDLC, high-density lipoprotein cholesterol; IFCC, International Federation of Clinical Chemistry and Laboratory Medicine; IgM, immunoglobulin; LDHI, lactate dehydrogenase according to IFCC; LDLC, low-density lipoprotein cholesterol; LIPC, lipase colorimetric; MG, magnesium; PHOS, phosphate; TP, total protein; TRIG, triglycerides; UA, uric acid; UREAL, urea/urea nitrogen.

**Supplementary Table 2** Summary of precision CVs for the c 703 analytical unit.

| Application | Control material | Site | Reproducibility CV (%) | Repeatability CV (%) | Intermediate CV (%) |
| --- | --- | --- | --- | --- | --- |
| ALB_2 | PC CC Multi 1 | 1 | 1.40 | 1.03 | 1.10 |
| ALB_2 | PC CC Multi 1 | 2 |  | 0.95 | 1.16 |
| ALB_2 | PC CC Multi 2 | 1 | 1.29 | 0.77 | 0.80 |
| ALB_2 | PC CC Multi 2 | 2 |  | 0.88 | 0.92 |
| ALBT_2 | PN PUC | 1 | 3.02 | 2.80 | 2.90 |
| ALBT_2 | PN PUC | 2 |  | 1.37 | 1.55 |
| ALBT_2 | PP PUC | 1 | 3.37 | 3.26 | 3.30 |
| ALBT_2 | PP PUC | 2 |  | 0.78 | 1.09 |
| ALP_2 | PC CC Multi 1 | 1 | 3.34 | 2.02 | 3.31 |
| ALP_2 | PC CC Multi 1 | 2 |  | 2.77 | 3.38 |
| ALP_2 | PC CC Multi 2 | 1 | 2.47 | 1.36 | 2.47 |
| ALP_2 | PC CC Multi 2 | 2 |  | 1.92 | 2.31 |
| ALTP_2 | PC CC Multi 1 | 1 | 2.62 | 1.84 | 1.84 |
| ALTP_2 | PC CC Multi 1 | 2 |  | 1.09 | 1.98 |
| ALTP_2 | PC CC Multi 2 | 1 | 1.87 | 0.97 | 1.26 |
| ALTP_2 | PC CC Multi 2 | 2 |  | 0.61 | 1.93 |
| APO AI | PC CC Multi 1 | 1 | 1.73 | 0.90 | 1.73 |
| APO AI | PC CC Multi 1 | 2 |  | 1.40 | 1.47 |
| APO AI | PC CC Multi 2 | 1 | 1.69 | 1.21 | 1.61 |
| APO AI | PC CC Multi 2 | 2 |  | 0.77 | 1.05 |
| ASTP_2 | PC CC Multi 1 | 1 | 1.70 | 1.39 | 1.60 |
| ASTP_2 | PC CC Multi 1 | 2 |  | 1.11 | 1.20 |
| ASTP_2 | PC CC Multi 2 | 1 | 1.54 | 0.62 | 0.75 |
| ASTP_2 | PC CC Multi 2 | 2 |  | 0.78 | 0.80 |
| Ca_2 | IQU Chem Con L1 | 1 | 2.26 | 1.35 | 1.36 |
| Ca_2 | IQU Chem Con L1 | 2 |  | 0.81 | 0.84 |
| Ca_2 | IQU Chem Con L2 | 1 | 1.89 | 1.16 | 1.19 |
| Ca_2 | IQU Chem Con L2 | 2 |  | 0.67 | 0.70 |
| Ca_2 | PC CC Multi 1 | 1 | 1.11 | 0.76 | 0.80 |
| Ca_2 | PC CC Multi 1 | 2 |  | 0.57 | 0.60 |
| Ca_2 | PC CC Multi 2 | 1 | 1.13 | 0.53 | 0.59 |
| Ca_2 | PC CC Multi 2 | 2 |  | 0.69 | 0.71 |
| CARB_4 | TDM CS L1 | 1 | 2.06 | 1.99 | 2.01 |
| CARB_4 | TDM CS L1 | 2 |  | 1.25 | 1.68 |
| CARB_4 | TDM CS L2 | 1 | 1.06 | 0.99 | 1.02 |
| CARB_4 | TDM CS L2 | 2 |  | 0.97 | 1.18 |
| CARB_4 | TDM CS L3 | 1 | 1.19 | 1.17 | 1.24 |
| CARB_4 | TDM CS L3 | 2 |  | 0.87 | 1.10 |
| CHE_2 | PC CC Multi 1 | 1 | 1.38 | 0.64 | 0.77 |
| CHE_2 | PC CC Multi 1 | 2 |  | 0.56 | 0.64 |
| CHE_2 | PC CC Multi 2 | 1 | 1.76 | 0.54 | 0.60 |
| CHE_2 | PC CC Multi 2 | 2 |  | 0.48 | 0.65 |
| CHOL_2 | PC CC Multi 1 | 1 | 1.69 | 0.92 | 1.28 |
| CHOL_2 | PC CC Multi 1 | 2 |  | 0.70 | 0.85 |
| CHOL_2 | PC CC Multi 2 | 1 | 1.27 | 0.73 | 0.92 |
| CHOL_2 | PC CC Multi 2 | 2 |  | 0.81 | 0.82 |
| CK | PC CC Multi 1 | 1 | 1.51 | 0.80 | 1.14 |
| CK | PC CC Multi 1 | 2 |  | 0.79 | 0.97 |
| CK | PC CC Multi 2 | 1 | 0.96 | 0.80 | 1.00 |
| CK | PC CC Multi 2 | 2 |  | 0.41 | 0.56 |
| CREJ_2 | IQU Chem Con L1 | 1 | 2.34 | 2.56 | 2.69 |
| CREJ_2 | IQU Chem Con L1 | 2 |  | 0.99 | 1.22 |
| CREJ_2 | IQU Chem Con L2 | 1 | 2.15 | 2.34 | 2.42 |
| CREJ_2 | IQU Chem Con L2 | 2 |  | 1.03 | 1.24 |
| CREJ_2 | PC CC Multi 1 | 1 | 3.02 | 3.11 | 3.44 |
| CREJ_2 | PC CC Multi 1 | 2 |  | 1.52 | 1.73 |
| CREJ_2 | PC CC Multi 2 | 1 | 2.50 | 2.69 | 2.91 |
| CREJ_2 | PC CC Multi 2 | 2 |  | 1.03 | 1.10 |
| CRP_4 | PC CC Multi 1 | 1 | 3.24 | 3.57 | 3.60 |
| CRP_4 | PC CC Multi 1 | 2 |  | 0.93 | 1.01 |
| CRP_4 | PC CC Multi 2 | 1 | 2.59 | 2.16 | 2.45 |
| CRP_4 | PC CC Multi 2 | 2 |  | 0.65 | 0.91 |
| Fe_2 | PC CC Multi 1 | 1 | 0.62 | 0.57 | 0.59 |
| Fe_2 | PC CC Multi 1 | 2 |  | 0.59 | 0.65 |
| Fe_2 | PC CC Multi 2 | 1 | 0.62 | 0.34 | 0.35 |
| Fe_2 | PC CC Multi 2 | 2 |  | 0.35 | 0.37 |
| GGT_2 | PC CC Multi 1 | 1 | 3.87 | 1.43 | 1.45 |
| GGT_2 | PC CC Multi 1 | 2 |  | 4.02 | 4.03 |
| GGT_2 | PC CC Multi 2 | 1 | 3.11 | 0.59 | 0.66 |
| GGT_2 | PC CC Multi 2 | 2 |  | 1.79 | 1.79 |
| GLUC_3 | IQU Chem Con L1 | 1 | 1.19 | 1.16 | 1.16 |
| GLUC_3 | IQU Chem Con L1 | 2 |  | 1.16 | 1.19 |
| GLUC_3 | IQU Chem Con L2 | 1 | 0.74 | 0.65 | 0.66 |
| GLUC_3 | IQU Chem Con L2 | 2 |  | 0.78 | 0.81 |
| GLUC_3 | PC CC Multi 1 | 1 | 0.90 | 0.62 | 0.65 |
| GLUC_3 | PC CC Multi 1 | 2 |  | 0.63 | 0.64 |
| GLUC_3 | PC CC Multi 2 | 1 | 0.63 | 0.41 | 0.45 |
| GLUC_3 | PC CC Multi 2 | 2 |  | 0.43 | 0.49 |
| HDL_4 | PC CC Multi 1 | 1 | 0.65 | 0.42 | 0.57 |
| HDL_4 | PC CC Multi 1 | 2 |  | 0.55 | 0.83 |
| HDL_4 | PC CC Multi 2 | 1 | 0.93 | 0.59 | 0.99 |
| HDL_4 | PC CC Multi 2 | 2 |  | 0.55 | 0.74 |
| IgM_2 | PC CC Multi 1 | 1 | 7.12 | 2.99 | 3.10 |
| IgM_2 | PC CC Multi 1 | 2 |  | 3.59 | 5.35 |
| IgM_2 | PC CC Multi 2 | 1 | 5.94 | 2.81 | 2.85 |
| IgM_2 | PC CC Multi 2 | 2 |  | 1.51 | 2.51 |
| LDHI_2 | PC CC Multi 1 | 1 | 1.58 | 1.68 | 1.73 |
| LDHI_2 | PC CC Multi 1 | 2 |  | 0.75 | 0.97 |
| LDHI_2 | PC CC Multi 2 | 1 | 1.55 | 1.59 | 1.59 |
| LDHI_2 | PC CC Multi 2 | 2 |  | 0.45 | 0.75 |
| LDLC_3 | PC CC Multi 1 | 1 | 1.45 | 1.02 | 1.07 |
| LDLC_3 | PC CC Multi 1 | 2 |  | 1.14 | 1.16 |
| LDLC_3 | PC CC Multi 2 | 1 | 1.21 | 0.77 | 0.82 |
| LDLC_3 | PC CC Multi 2 | 2 |  | 0.77 | 0.86 |
| LIPC | PC CC Multi 1 | 1 | 4.54 | 2.24 | 3.90 |
| LIPC | PC CC Multi 1 | 2 |  | 0.94 | 1.24 |
| LIPC | PC CC Multi 2 | 1 | 4.90 | 2.66 | 4.48 |
| LIPC | PC CC Multi 2 | 2 |  | 0.55 | 0.99 |
| MG_2 | IQU Chem Con L1 | 1 | 1.48 | 1.17 | 1.25 |
| MG_2 | IQU Chem Con L1 | 2 |  | 1.04 | 1.31 |
| MG_2 | IQU Chem Con L2 | 1 | 1.39 | 1.34 | 1.38 |
| MG_2 | IQU Chem Con L2 | 2 |  | 0.54 | 0.63 |
| MG_2 | PC CC Multi 1 | 1 | 1.08 | 0.55 | 0.71 |
| MG_2 | PC CC Multi 1 | 2 |  | 0.40 | 0.48 |
| MG_2 | PC CC Multi 2 | 1 | 1.23 | 0.74 | 0.94 |
| MG_2 | PC CC Multi 2 | 2 |  | 0.34 | 0.39 |
| PHOS_2 | IQU Chem Con L1 | 1 | 1.34 | 1.34 | 1.36 |
| PHOS_2 | IQU Chem Con L1 | 2 |  | 1.12 | 1.21 |
| PHOS_2 | IQU Chem Con L2 | 1 | 1.06 | 1.13 | 1.14 |
| PHOS_2 | IQU Chem Con L2 | 2 |  | 0.73 | 0.80 |
| PHOS_2 | PC CC Multi 1 | 1 | 0.76 | 0.58 | 0.60 |
| PHOS_2 | PC CC Multi 1 | 2 |  | 0.57 | 0.62 |
| PHOS_2 | PC CC Multi 2 | 1 | 0.91 | 0.44 | 0.46 |
| PHOS_2 | PC CC Multi 2 | 2 |  | 0.44 | 0.44 |
| TP_2 | PC CC Multi 1 | 1 | 0.89 | 0.87 | 0.96 |
| TP_2 | PC CC Multi 1 | 2 |  | 0.40 | 0.57 |
| TP_2 | PC CC Multi 2 | 1 | 1.11 | 0.59 | 0.69 |
| TP_2 | PC CC Multi 2 | 2 |  | 0.31 | 0.35 |
| TRIG | PC CC Multi 1 | 1 | 1.08 | 1.05 | 1.16 |
| TRIG | PC CC Multi 1 | 2 |  | 0.52 | 0.75 |
| TRIG | PC CC Multi 2 | 1 | 0.78 | 0.65 | 0.73 |
| TRIG | PC CC Multi 2 | 2 |  | 0.66 | 0.68 |
| UA_2 | IQU Chem Con L1 | 1 | 1.84 | 1.34 | 2.02 |
| UA_2 | IQU Chem Con L1 | 2 |  | 1.29 | 1.34 |
| UA_2 | IQU Chem Con L2 | 1 | 1.50 | 1.06 | 1.63 |
| UA_2 | IQU Chem Con L2 | 2 |  | 0.78 | 0.82 |
| UA_2 | PC CC Multi 1 | 1 | 1.37 | 0.76 | 1.33 |
| UA_2 | PC CC Multi 1 | 2 |  | 0.51 | 0.68 |
| UA_2 | PC CC Multi 2 | 1 | 1.65 | 0.73 | 1.51 |
| UA_2 | PC CC Multi 2 | 2 |  | 0.70 | 0.76 |
| UREAL | IQU Chem Con L1 | 1 | 3.67 | 3.82 | 3.99 |
| UREAL | IQU Chem Con L1 | 2 |  | 2.95 | 3.00 |
| UREAL | IQU Chem Con L2 | 1 | 2.35 | 2.30 | 2.40 |
| UREAL | IQU Chem Con L2 | 2 |  | 1.77 | 1.86 |
| UREAL | PC CC Multi 1 | 1 | 1.87 | 1.39 | 1.65 |
| UREAL | PC CC Multi 1 | 2 |  | 0.98 | 1.15 |
| UREAL | PC CC Multi 2 | 1 | 1.59 | 0.74 | 1.40 |
| UREAL | PC CC Multi 2 | 2 |  | 0.66 | 1.02 |

ALB, albumin; ALBT, Tina-quant albumin; ALP, alkaline phosphatase; ALTP, alanine aminotransferase according to IFCC; APO AI, apolipoprotein A‑1; ASTP, aspartate aminotransferase according to IFCC; Ca, calcium; CARB, carbamazepine; CHE, cholinesterase; CHOL, cholesterol; CK, creatine kinase; CREJ, creatinine Jaffé; CRP, C-reactive protein; CV, coefficient of variation; Fe, iron; GGT, γ-glutamyltransferase; GLUC, glucose; HDL, high-density lipoprotein; IFCC, International Federation of Clinical Chemistry and Laboratory Medicine; IgM, immunoglobulin; LDHI, lactate dehydrogenase according to IFCC; LDLC, low-density lipoprotein cholesterol; LIPC, lipase colorimetric; MG, magnesium; PHOS, phosphate; TP, total protein; TRIG, triglycerides; UA, uric acid; UREAL, urea/urea nitrogen.

**Supplementary Fig. 1** Passing-Bablok regression and Bland-Altman plots of the two applications with the highest bias in slope (**A**; CRP) and intercept (**B**; CHE).

**A)**


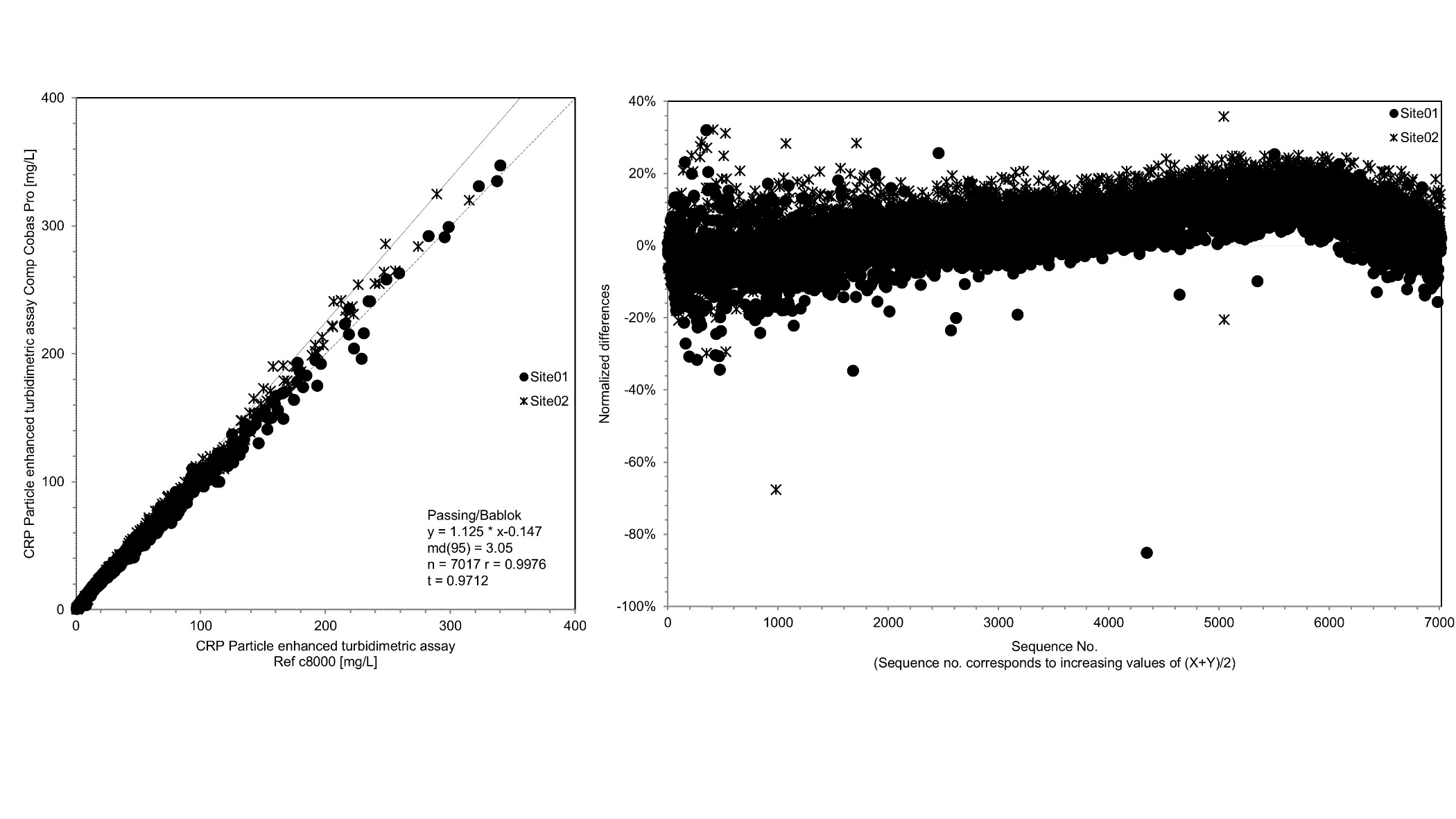


**B)**


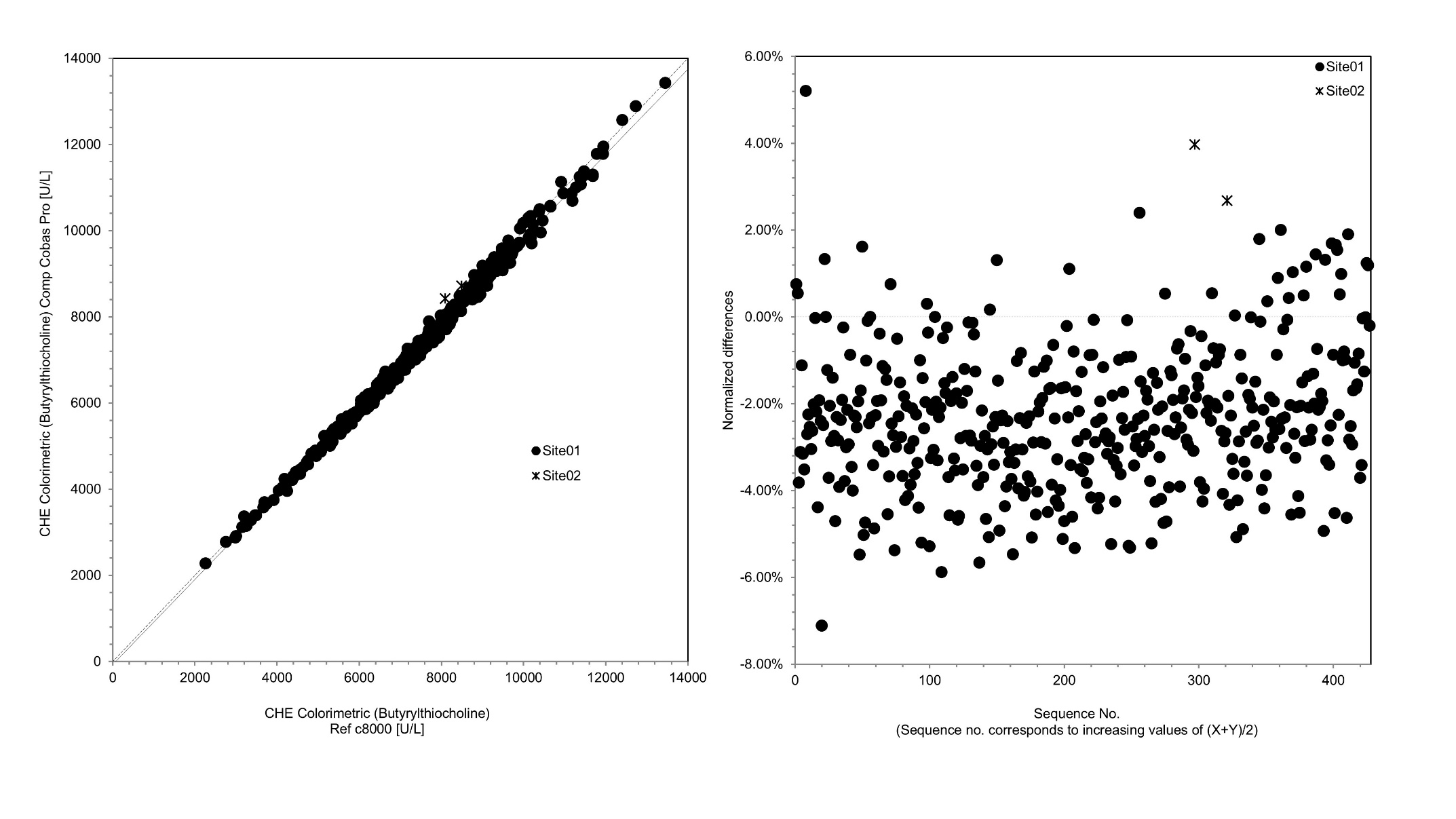


CHE, cholinesterase; CRP, C-reactive protein.
